# Increases of Obesity among Iranian Adults: An Alarm for Policymakers

**Published:** 2020-04

**Authors:** Salman KHAZAEI, Saeid BASHIRIAN, Ensiyeh JENABI

**Affiliations:** 1. Research Center for Health Sciences, Hamadan University of Medical Sciences, Hamadan, Iran; 2. Social Determinants of Health Research Center, Hamadan University of Medical Sciences, Hamadan, Iran; 3. Autism Spectrum Disorders Research Center, Hamadan University of Medical Sciences, Hamadan, Iran

## Dear Editor in Chief

Obesity is one of the major public health problems of the twenty-first century (1). The continuation of obesity lead to increased morbidity risk in later life such as diabetes, coronary heart disease (CHD) and some of cancers (1–3). The prevalence of obesity has been elevating rapidly all round the world and is estimated to elevate further (4). Weight increases from imbalance of positive energy that calories expended more than those consumed to support basal metabolism, activity and growth, although the etiology of obesity is multi-factorial (5). Iran is one of the countries with the high prevalence of obesity among adults. Therefore, we elevated the prevalence and trend of obesity among Iranian adults (6).

**Fig. 1:**
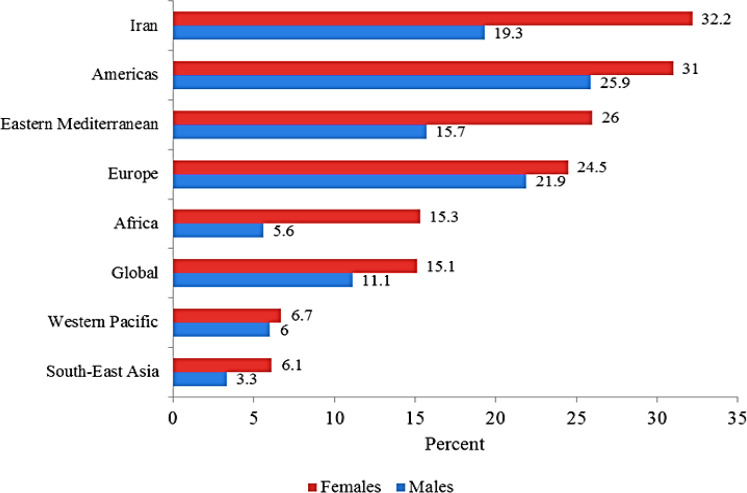
Prevalence of obesity among adults (%), BMI≥ 30, age-standardized estimates by WHO region in 2016

Figure 1 shows the prevalence of obesity among adults in Iran compared the other WHO regions in 2016.

The highest prevalence of obesity among both males and females are found in Americas region (25.9% and 31% respectively). While South East Asia countries with the 6.1% prevalence rate for females and 3.3% for males had the lowest rate. The prevalence rate of obesity in Iran was higher than all regions for females (32.2%). Except Americas region and prevalence rate of obesity for Iranian men was higher than other WHO regions (19.3%), which was much higher than global average (15.1% for females and 11.1% for males) for both males and females.

**Fig. 2:**
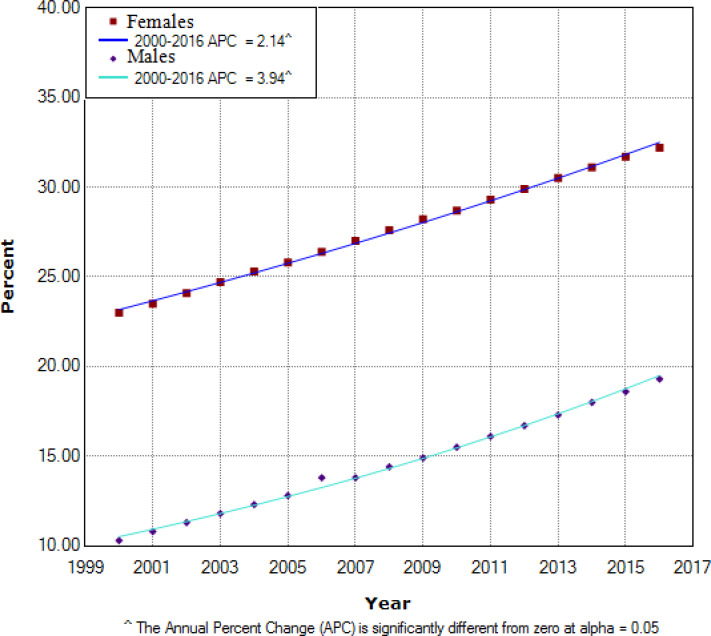
Trend analysis for prevalence of obesity among Iranian adults (BMI**≥**30) (2000–2016)

As shown in Fig. 2, trend of obesity for both males and females was increasing from 2000 to 2016. For males, prevalence of obesity among adults reached from 10.3% in 2000 to 19.3% in 2016 (Annual percent change (APC): 3.94%, *P*<0.05), while for females reached from 19.3% in 2000 to 32.2% in 2016 (Annual percent change (APC): 2.14%, *P*<0.05). The reason of obesity among Iranian adults is patterns of inappropriate dietary habits, using of junk food and limitation of physical activity due to society’s modernization. There is a need to establish programs for elevating awareness among the population in related to the obesity complications, control methods of obesity and performing of organized educational and preventive programs in adults.
